# Enzymatically Cross-Linked Hydrogel Beads Based on a Novel Poly(aspartamide) Derivative

**DOI:** 10.3390/gels11020093

**Published:** 2025-01-26

**Authors:** Wenzhuo Hou, Hui Yi, Guangyan Zhang

**Affiliations:** 1 School of Materials and Chemical Engineering, Hubei University of Technology, Wuhan 430068, China; houwenzhuohbut@163.com (W.H.); yihuihbut@163.com (H.Y.); 2 Hubei Provincial Key Laboratory of Green Materials for Light Industry, Hubei University of Technology, Wuhan 430068, China

**Keywords:** poly(aspartamide) derivative, in situ hydrogel, injectable hydrogel, hydrogel beads, horseradish peroxidase, enzyme-catalyzed cross-linking

## Abstract

In recent years, hydrogel beads and in situ hydrogels have gained wide attention in various fields such as biomedicine. In this study, 3-(4-hydroxyphenyl) propionic acid (HP) was introduced into the side chain of poly(α,β-[*N*-(2-hydroxyethyl)-D,L-aspartamide]) (PHEA) to synthesize phenolic hydroxyl-functionalized poly(aspartamide) derivative PHEA-HP with enzyme-catalyzed cross-linking potential. First, the chemical structure of PHEA-HP was characterized by FT-IR, UV and ^1^H NMR, and the results of in vitro cytotoxicity against L929 cell line and hemolysis experiment showed that PHEA-HP did not have toxicity to cells (viability > 90%) and had good blood compatibility. Then, rheological measurement confirmed the formation of PHEA-HP-based in situ hydrogel with a high storage modulus (G′) around 10^4^ Pa, and the vial-tilting method revealed that the gelation time of PHEA-HP aqueous solution could be tuned in the wide range of 5–260 s by varying the concentrations of hydrogen peroxide (H_2_O_2_) and horseradish peroxidase (HRP). Finally, hydrogel beads of different diameters containing methylene blue (for easy observation) were prepared using a coaxial needle and syringe pumps, and the effect of the flow rate of the outer phase on the diameters of the hydrogel beads was also investigated. Therefore, PHEA-HP may be a promising and safe poly(aspartamide) derivative that can be used to prepare in situ hydrogels and hydrogel beads for applications closely related to the human body.

## 1. Introduction

Hydrogel beads are extensively studied as encapsulation media for biomedicine, food, and pharmaceutical applications or as adsorption systems in the field of environmental protection. In the biomedical field, hydrogel beads are often used as drug carriers to achieve sustained or controlled release of drugs [1,2,3]. In the food field, the potential health benefits of many nutraceuticals have not yet been fully realized due to chemical degradation during storage or in the gastrointestinal tract. Hydrogel beads can improve nutraceutical performance by protecting them from chemical degradation [4,5]. In addition, enzymes also can be encapsulated in hydrogel beads to improve their utilization and activity in foods [6]. In the field of environmental protection, hydrogel beads can be used to adsorb and remove organic pollutants from wastewater, including dyes [7], active pharmaceutical ingredients [8], heavy metals [9], and pesticides [10,11,12].

Common materials for preparing hydrogel beads include difficult-to-degrade carbon chain polymers (e.g., poly(vinyl alcohol) (PVA) [13], poly(*N*-isopropylacrylamide) [14]) and degradable polymers (e.g., alginate [15,16], chitosan [17,18,19] and cellulose [20]). Although the carbon-carbon backbone is beneficial to the stability of the hydrogel beads, it also brings adverse impacts on the environment and health. As people’s awareness of environmental protection and health increases, the use of biodegradable polymers to prepare hydrogel beads has attracted widespread attention, especially in the fields of biomedicine, food, and cosmetic that are closely related to human health.

To prepare hydrogel beads, cross-linking is a necessary process because cross-linking can transform the polymer solution from a liquid state to a “gel” or “solid” state by restricting the mobility of the molecular chains. Cross-linking can be ionic or covalent. The typical ionic crosslinking systems include alginate/Ca^2+^ [21,22]. For example, curcumin, a natural polyphenolic product with multiple physiological activities such as antioxidant and anti-inflammatory, was encapsulated into hydrogel beads made of alginate/Ca^2+^ [23]. For covalent cross-linking, chemical cross-linkers are the most commonly used method. For instance, boric acid is often used as a cross-linker for preparing PVA hydrogel or hydrogel beads [24]. In addition to cross-linkers, enzymes such as horseradish peroxidase (HRP) have also been widely employed to prepare hydrogels, especially in situ hydrogels and injectable hydrogels [25,26]. Importantly, their gelation time can be tuned by the concentration of enzymes [27], which is of great significance in practical applications. In situ hydrogels and injectable hydrogels have not only been intensively studied in applications such as wound repair [28,29,30], tissue engineering [31,32,33], and flexible microrobots [34], but also provide the possibility for the preparation of hydrogel beads.

Poly(aspartamide) derivatives are a class of polymers with a backbone consisting of aspartic acid, and can be biodegraded by the hydrolysis of the amide linkages on their backbone [35]. Over the past few decades, the interest in poly(aspartamide) derivatives has increased significantly in various fields, especially in the biomedical field, not only because of their good biodegradability but also because of their good safety profile in humans [36]. The enzyme-mediated cross-linking injectable hydrogels based on poly(aspartamide) derivatives are one of them [37]. However, there is no report on the preparation of regular hydrogel beads using enzymatically cross-linkable poly(aspartamide) derivatives.

Poly(α,β-[*N*-(2-hydroxyethyl)-D,L-aspartamide]) (PHEA), one of the most investigated poly(aspartamide) derivatives, can be easily prepared via the aminolysis ring-opening reaction of poly(succinimide) (PSI) with ethanolamine in various solvents (e.g., *N*,*N*-dimethylformamide (DMF), dimethylsulphoxide) under mild reaction conditions. Since the side chain of PHEA is rich in hydroxyl groups and is easily modified, molecules with different groups or functions have been introduced into PHEA for cancer therapy [38], drug carriers [39], siRNA delivery [40], and oral peptide/protein delivery [41]. Therefore, it may be interesting work to introduce phenolic hydroxyl groups into PHEA to cause it to have the potential for enzymatic cross-linking, and then use it to prepare hydrogel beads. In this study, phenolic hydroxyl-functionalized PHEA, denoted as PHEA-HP, was designed. Firstly, the preparation, structural characterization, and gelation of PHEA-HP were studied, and the cytotoxicity of PHEA-HP was also evaluated. Then, the effects of HRP and hydrogen peroxide (H_2_O_2_) concentrations on the gelation time were studied. Finally, PHEA-HP-based hydrogel beads containing methylene blue (for easy observation) were prepared using a coaxial needle and two syringe pumps.

## 2. Results and Discussion

### 2.1. Synthesis and Characterization of PHEA-HP

**Scheme 1:**
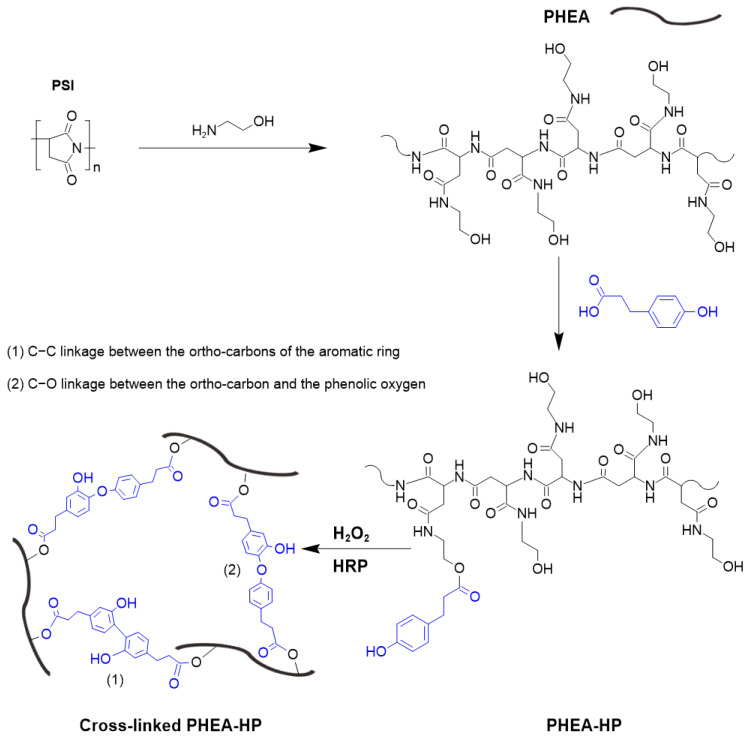
Synthesis route and cross-linking mechanism of PHEA-HP.

The synthetic route of PHEA-HP is illustrated in Scheme 1. First, poly(α,β-[*N*-(2-hydroxyethyl)-D,L-aspartamide]) (PHEA) was prepared from PSI with excess ethanolamine. Then, PHEA was esterified with 3-(4-hydroxyphenyl) propionic acid (HP) to obtain phenolic hydroxyl-functionalized poly(aspartamide) derivative PHEA-HP.

**Figure 1:**
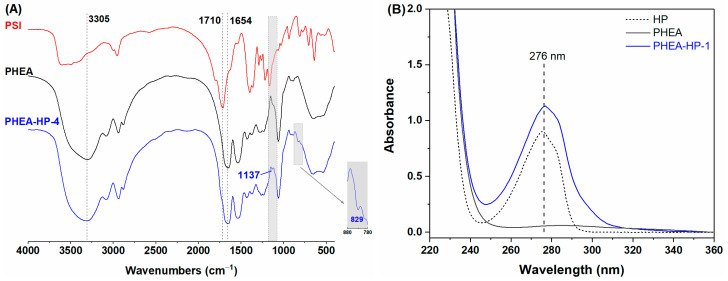
(**A**) FT-IR spectra of PSI, PHEA, and PHEA-HP-4 in KBr, and (**B**) UV spectra of HP (0.1 mg/mL), PHEA (2.0 mg/mL), and PHEA-HP-1 (2.0 mg/mL) in deionized water.

A series of PHEA-HP were synthesized by varying the molar feed ratio of HP to the polymer units of PHEA, as shown in Table 1. The structure of the obtained PHEA-HP was qualitatively analyzed by FT-IR (Figure 1A). It can be seen from Figure 1A that PHEA can be distinguished from PSI clearly. Compared with the spectrum of PSI, a new wide peak was observed at 3305 cm^−1^, which can be ascribed to the N–H stretch and introduced -OH. Additionally, the peak corresponding to the stretching vibrations of C=O (imide group in PSI, 1710 cm^−1^) also shifted to 1654 cm^−1^ (CONH in PHEA) after PSI was aminolyzed by ethanolamine. Moreover, the chemical structure of PHEA was further confirmed by ^1^H NMR (Figure S1) in D_2_O. The molecular weight and the molecular weight distribution (MWD) of the PHEA were 50.4 kDa and 1.30, respectively.

Compared with the FT-IR spectrum of PHEA, a new absorption peak of PHEA-HP-4 was detected at 1137 cm^−1^, which corresponds to the C–O–C of the ester bond formed by HP and PHEA. In addition, the new absorption peak corresponding to the C–H out-of-plane bending vibrations of HP moieties in PHEA-HP-4 was also observed at 829 cm^−1^. The FT-IR spectra of PHEA-HP-2 and PHEA-HP-3 (Figure S2) were very similar to that of PHEA-HP-4. However, the FT-IR spectrum of PHEA-HP-1 (Figure S2) was very similar to that of PHEA, no obvious absorption peaks were observed at both 1137 cm^−1^ and 829 cm^−1^, which may be attributed to the relatively low amount of HP moieties introduced. Therefore, PHEA-HP-1 was further analyzed by UV spectroscopy.

The UV spectra of HP, PHEA, and PHEA-HP-1 are shown in Figure 1B. It can be seen that a strong UV absorbance was observed at 276 nm for PHEA-HP-1, which corresponds to the UV absorption peak of HP, while PHEA has no obvious UV absorbance at 276 nm. It indicates that HP moieties were also contained in PHEA-HP-1. The results of FT-IR and UV indicate that HP moieties had been introduced into all four PHEA-HPs.

**Figure 2:**
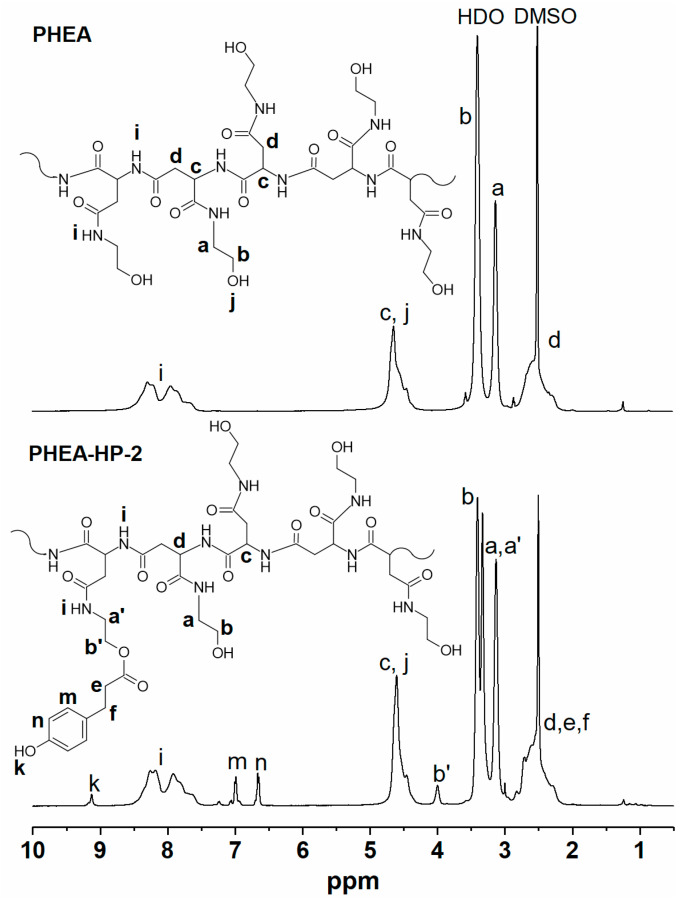
^1^H NMR spectra of PHEA and PHEA-HP-2 in DMSO-*d*_6_.

Quantitative analysis was further performed by ^1^H NMR for all four PHEA-HPs. As shown in Figure 2, compared with the ^1^H NMR spectrum of PHEA, four new peaks appeared at 4.0 ppm (peak b’), 6.6 ppm (peak n), 7.0 ppm (peak m), and 9.2 ppm (peak k) in PHEA-HP-2. The proton hydrogen signal peak of the -CH_2_- adjacent to the -OH on the PHEA side chains shifted from 3.4 ppm (peak b) to 4.0 ppm (peak b’), indicating that ester linkages have been formed between PHEA and HP. Peaks n and m can be attributed to the proton hydrogen signals of the benzene ring on HP moieties, and peak k indicates that the phenolic hydroxyl group has been successfully introduced. The percentage of introduced HP to the polymer units of PHEA in four PHEA-HPs was calculated based on the ratio of the integral of peak n (6.6 ppm, 2H in HP moieties) to the integral of peak a and a’ (3.1 ppm, 2H in PHEA). The results are summarized in Table 1.

According to the results of FT-IR, UV, and ^1^H NMR, it can be concluded that PHEA-HP has been successfully synthesized. However, the reaction efficiency between HP and PHEA was very low (<20%, Table 1). When the feed ratio of HP was greater than 50%, increasing the feed ratio of HP could not effectively increase the HP moieties introduced into PHEA-HP. Therefore, PHEA-HP-3 and PHEA-HP-4 were not evaluated further, although they were also able to form in situ hydrogels.

### 2.2. Hydrogel Formation

**Figure 3:**
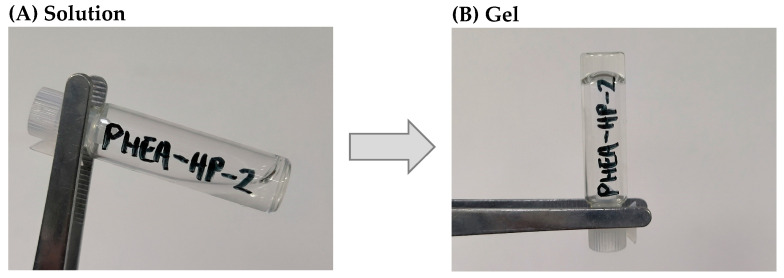
Photographs of PHEA-HP-2/HRP/H_2_O_2_ aqueous solution (**A**) before and (**B**) after gelation.

When two equivalent volumes of PHEA-HP aqueous solutions containing either HRP (solution A) or H_2_O_2_ (solution B) were mixed, and a pale yellow transparent in situ hydrogel was rapidly formed as shown in Figure 3. Although both PHEA-HP-1 and PHEA-HP-2 could form in situ hydrogels in the presence of H_2_O_2_ and HRP, PHEA-HP-1 formed hydrogel significantly more slowly and weakly than PHEA-HP-2, which may be ascribed to the lower percentage of HP moieties in PHEA-HP-1.

**Figure 4:**
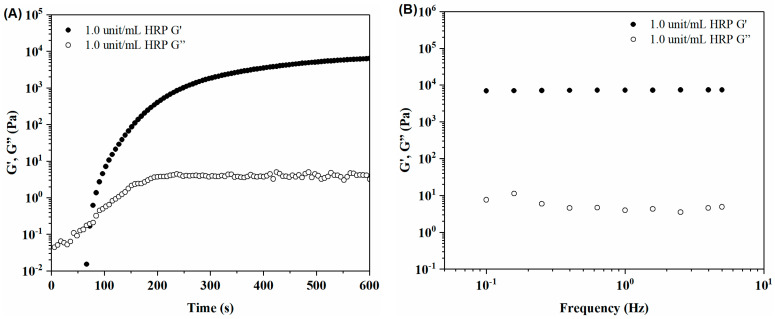
(**A**) The storage modulus (G′) and loss modulus (G″) of PHEA-HP-2/HRP/H_2_O_2_ solution as a function of time, and (**B**) frequency dependencies of the G′ and G″ of PHEA-HP-2/HRP/H_2_O_2_ in situ hydrogel at 25 °C (PHEA-HP-2: 6.0 wt.%, HRP: 1.0 unit/mL, H_2_O_2_: 5.0 mM).

Rheological measurement was further performed to monitor the solution-gel transition of PHEA-HP aqueous solutions at 25 °C. Since the gelation rate was very fast, when two equal volumes of PHEA-HP/HRP solution and PHEA-HP/H_2_O_2_ solution were mixed and transferred to the parallel plate of the rheometer, the hydrogel was formed immediately and exhibited a storage modulus (G′) greater than loss modulus (G″) at the beginning of the rheological test. Therefore, the sample (PHEA-HP-2: 6.0 wt.%, HRP: 1.0 unit/mL, H_2_O_2_: 5.0 mM) with a long gelation time was chosen as representative to explain the solution-gel transition of PHEA-HP-2 aqueous solution (Figure 4A).

As shown in Figure 4A, at the beginning of the test, G′ and G″ were both very low (<0.2 Pa), and G′ was lower than G″. As time went on, the crossover of G′ and G″ was observed at 72 s. Since the crossover point was defined as the gelation point [42], it was confirmed that the solution–gel transition occurred. Subsequently, G′ strengthened rapidly and finally reached nearly 10^4^ Pa at the end of the test (600 s), while G″ increased slowly and eventually reached a plateau of only around 5 Pa. This indicates that the hydrogel has been fully formed, and the changing trends of G′ and G″ are consistent with those in other studies [37,43]. Figure 4B showed the frequency scanning curves of the hydrogel formed from the sample (PHEA-HP-2: 6.0 wt.%, HRP: 1.0 unit/mL, H_2_O_2_: 5.0 mM) with a scanning frequency range from 0.01 Hz to 5.0 Hz. Both G′ and G″ of the in situ hydrogel remained stable, indicating that the frequency had little effect on the strength of the hydrogel.

Compared with PHEA-HP-2, due to the less HP moieties introduced into PHEA-HP-1, most of the PHEA-HP-1-based in situ hydrogels with long gelation time (>30 s) had significantly lower G′ (10–100 Pa, Figure S3) than that of PHEA-HP-2-based in situ hydrogels (10^4^ Pa). Therefore, PHEA-HP-2 was preferred for preparing hydrogel beads and further studies were conducted.

### 2.3. In Vitro Cytotoxicity and Hemolysis Experiment

**Figure 5:**
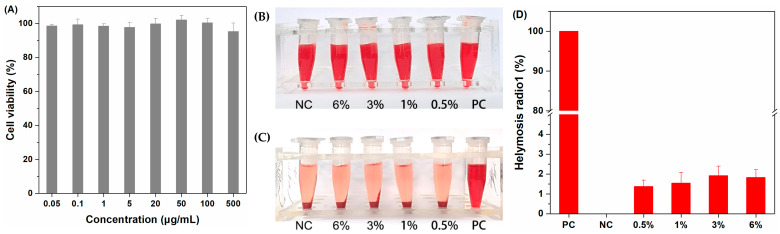
(**A**) In vitro cytotoxicity of PHEA-HP-2 against L929 cells after 48 h incubation (mean ± SD, *n* = 4), the hemolysis experiment (**B**) before and (**C**) after centrifuge, and (**D**) the hemolysis ratio of PHEA-HP-2 with various concentrations.

The safety of PHEA-HP-2 was evaluated through an in vitro cytotoxicity assay on L929 cells using CCK-8 method. As shown in Figure 5, the cell viability of L929 cells was higher than 90% at all tested polymer concentrations ranging from 0.05–500 μg/mL, indicating that PHEA-HP-2 did not show significant cytotoxicity to L929 cells even at the highest concentration of 500 μg/mL.

In addition to in vitro cytotoxicity, a hemolysis experiment was also performed to evaluate the hemocompatibility of PHEA-HP-2, as they may be used in applications that contact with blood, such as wound dressings. As shown in Figure 5B,C, at all PHEA-HP-2 concentrations tested (0.5–6.0%), the red blood cells (RBC) aggregated at the bottom of the solution, similar to the negative control (PBS buffer, NC); while the positive control (1% Triton X-100, PC) solution remained bright red before and after centrifugation. The supernatants were scanned at 540 nm to determine the hemolysis ratio and all tested concentrations of PHEA-HP-2 showed good blood compatibility (<3%), as shown in Figure 5D.

### 2.4. Gelation Time

For preparing hydrogel beads using a coaxial needle and syringe pumps, gelation time is an important parameter. If the gelation time is too long, the formed hydrogel beads tend to stick together in the collection container. If the gelation time is too short, the mixer and coaxial needle may become clogged. Therefore, the gelation time was investigated before preparing the hydrogel beads.

**Figure 6:**
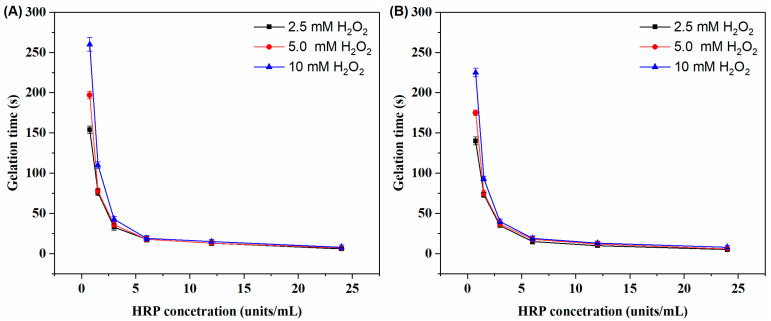
Gelation time of PHEA-HP-2 hydrogels in deionized water as a function of the concentration of HRP and H_2_O_2_: (**A**) 3.0 wt.% PHEA-HP-2 and (**B**) 6.0 wt.% PHEA-HP-2; three replicates were performed for each experiment.

The effects of HRP, H_2_O_2_, and PHEA-HP-2 concentrations on gelation time were investigated by the vial-tilting method instead of rheometry due to their high gelation rate. As shown in Figure 6, the gelation time of PHEG-HP-2 hydrogels can be easily tuned in a wide range from 5 to 260 s by varying the concentrations of HRP (0.75–24 units/mL), H_2_O_2_ (2.5–10 mM), or PHEA-HP-2 (3.0 wt.% and 6.0 wt.%).

When the concentration of PHEA-HP-2 was fixed at 3.0 wt.% (Figure 6A) or 6.0 wt.% (Figure 6B), the gelation time became shorter as the HRP concentration increased, and when the HRP concentration was higher than or equal to 6.0 units/mL, the gelation time was less than 20 s. On the other hand, as the H_2_O_2_ concentration increased, the gelation time became longer which was particularly obvious at the HRP concentration of 0.75 mM. The trend of gelation time with HRP and H_2_O_2_ is in correlation with the previous report [37]. In addition, at the same HRP and H_2_O_2_ concentrations, the gelation time of the hydrogel with higher PHEA-HP-2 content (6.0 wt.%, Figure 6B) was slightly shorter than that of the hydrogel with lower PHEG-HP-2 content (3.0 wt.%, Figure 6A). Therefore, the HRP concentration is an optimal and easily adjustable parameter to tune the gelation time. From the above results, it can be seen that HRP concentration is a key parameter for regulating gelation time. Moreover, at low HRP concentration, H_2_O_2_ concentration is also crucial to the regulation of gelation time.

### 2.5. Preparation of PHEA-HP-Based Hydrogel Beads

**Scheme 2:**
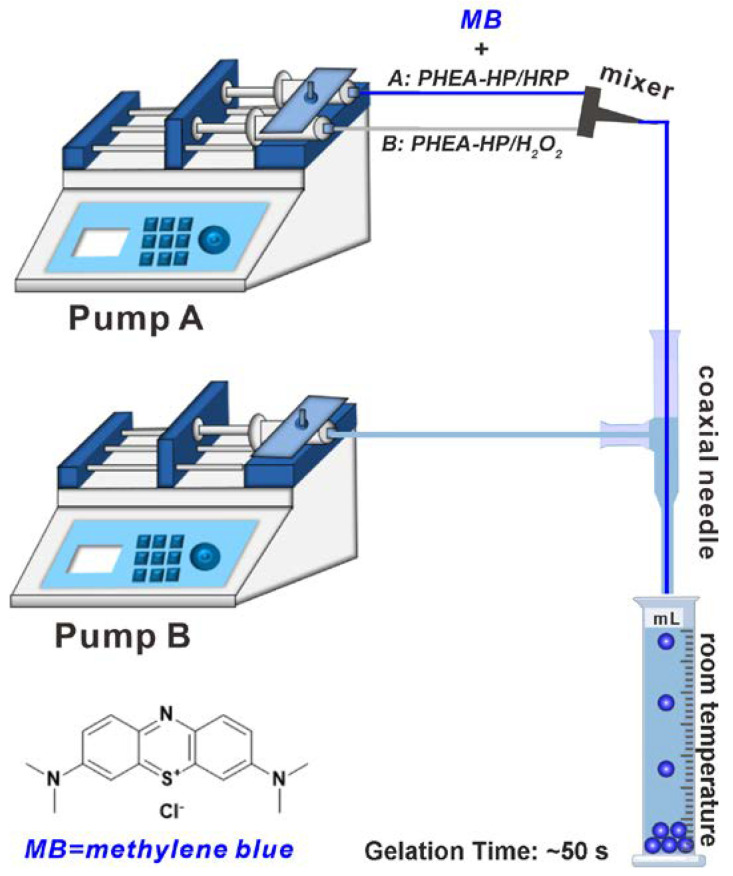
The formation of PHEA-HP-based hydrogel beads containing MB.

PHEA-HP-based hydrogel beads containing methylene blue (for easy observation) were prepared using a coaxial needle and two syringe pumps, as depicted in Scheme 2. Taking into account the time it takes to flow into the coaxial needle after the solutions A and B are mixed in the mixer, as well as the time required for the sedimentation process in dimethicone, a formula (PHEA-HP-2: 6.0 wt.%, HRP: 2.5 units/mL, H_2_O_2_: 5.0 mM) with a gelation time of about 50 s was selected for the preparation of hydrogel beads. Compared with other oils (e.g., vegetable oil), the density of dimethicone (0.96–0.98) is closer to that of water, so it has a relatively long sedimentation time, allowing PHEA-HP to completely gel. Thus, dimethicone was selected as the outer phase and collection medium. In addition, after the balls formed by the coaxial needle enter the dimethicone in the collection container, the viscosity of the dimethicone also has an important influence on whether they can form regular spheres through gelation. Thus, dimethicones with different viscosities (20, 100, and 1000 mPa∙s) were used to further control the sedimentation time to complete the enzymatic cross-linking to form hydrogel beads.

**Figure 7:**
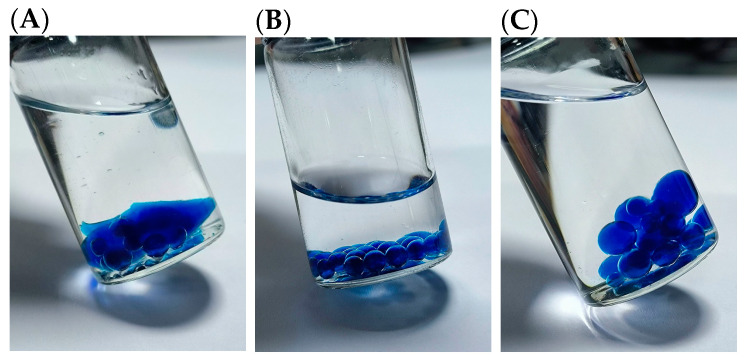
Photographs of the formed hydrogel beads in dimethicone with various viscosities: (**A**) 20 mPa∙s, (**B**) 100 mPa∙s, and (**C**) 1000 mPa∙s (pump A: 320 μL/min; pump B: 320 μL/min).

In the dimethicone with a viscosity of 20 mPa∙s, due to the rapid sedimentation rate, the small balls formed by the coaxial needle had not yet completely gelated when they reached the bottom of the collection container. Therefore, they stuck to each other and could not even maintain a spherical shape, but formed a whole piece (Figure 7A). In the dimethicone with a viscosity of 100 mPa∙s, the small balls completed gelation before reaching the bottom of the container, so independent, regular, spherical hydrogel beads can be obtained (Figure 7B). In the dimethicone with a viscosity of 1000 mPa∙s, the small balls adhered to each other before completing gelation and formed larger balls in the upper layer of dimethicone in the collection container, and finally obtained hydrogel beads with larger and non-uniform sizes (Figure 7C).

**Figure 8:**
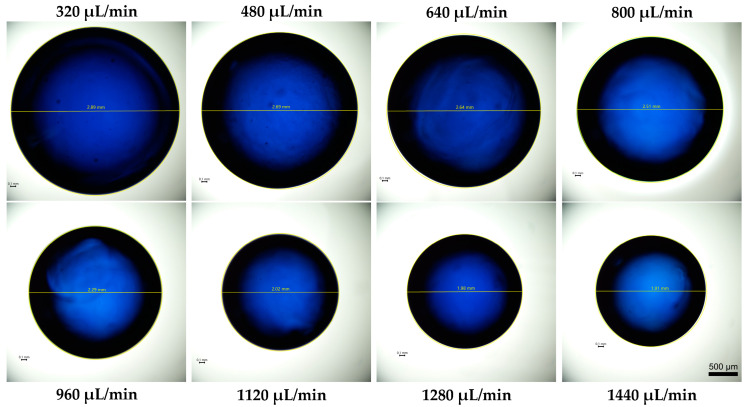
The optical images of MB-loaded hydrogel beads at different Q_o_ (the flow rate of pump A: 320 μL/min; the viscosity of dimethicone: 100 mPa∙s).

**Figure 9:**
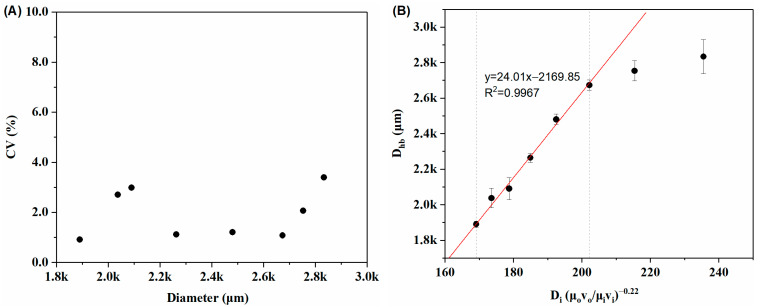
(**A**) Coefficient of variation (CV) values for the hydrogel beads with different average diameters. (**B**) Variation in the diameters of hydrogel beads (D_hb_) corresponds to D_i_(μ_o_v_o_/μ_i_v_i_)^−0.22^ (the solid red line is the linear fitting line).

The effect of Q_o_ (the flow rate of the outer continuous phase, pump B) on the size of the hydrogel beads was also investigated. As shown in Figure 8, when the flow rate of pump A (inner dispersed phase) was fixed at 320 μL/min, MB-loaded hydrogel beads with regular spherical shapes could be prepared in the range of Q_o_ from 320 μL/min to 1440 μL/min, and the diameters of the obtained hydrogel beads decreased with increasing the flow rate of the outer continuous phase. The coefficient of variation (CV) of MB-loaded hydrogel beads was calculated with Equation (1) [44]:

(1) $$CV=\frac{\sigma }{D_{hb}}$$

where σ is the standard deviation and D_hb_ is the average diameters of hydrogel beads. It can be seen from Figure 9A that the CV values of MB-loaded hydrogel beads are less than 5%, which indicates that the hydrogel beads have a uniform size.

The effect of the velocity of the outer phase (v_o_) on the size of the hydrogel beads was also further investigated by the empirical Equation (2) [44,45]:

(2) $$D_{hb}=D_{i}\times K{\left(\frac{\mu_{o}v_{o}}{\mu_{i}v_{i}}\right)}^{-0.22}+\alpha$$

where D_i_ is the inner diameter of the inner dispersed phase tube (400 μm), μ_o_ is the viscosity of the outer phase (dimethicone: 100 mPa∙s), μ_i_ is the viscosity of the inner phase (6.0 wt.% PHEA aqueous solution), v_o_ and v_i_ are the velocity of the outer phase and inner phase, respectively.

As shown in Figure 9B, D_hb_ and D_i_(μ_o_v_o_/μ_i_v_i_)^−0.22^ showed a good linear relationship with R^2^ of 0.9967 in the range of Q_o_ from 640 μL/min to 1440 μL/min. The obtained values for K and α in Equation (2) are 24.01 and −2169.85, respectively. However, when Q_o_ was 320 and 480 μL/min, D_hb_ and D_i_(μ_o_v_o_/μ_i_v_i_)^−0.22^ did not conform to empirical Equation (2). This may be related to the fact that when the flow rate of the outer phase was low, the inner and outer phases formed droplets at the outlet of the coaxial needle, and then dripped into dimethicone drop by drop. Since it was not a continuous flow at that time, it did not conform to Equation (2). Although PHEA-HP-2 can be prepared into hydrogel beads by the in situ gelation method, the low reaction efficiency between HP and PHEA may limit its large-scale production, and the stability of these hydrogel beads in different environments also needs to be further studied in practice.

## 3. Conclusions

In this work, a novel phenolic hydroxyl-functionalized poly(aspartamide) derivative, PHEA-HP, was successfully synthesized via the esterification reaction between 3-(4-hydroxyphenyl) propionic acid and poly(α,β-[*N*-(2-hydroxyethyl)-D,L-aspartamide]). PHEA-HP aqueous solution can undergo enzymatic cross-linking to form in situ hydrogels in the presence of H_2_O_2_ and HRP, and the gelation time can be tuned in the range of 5–260 s by adjusting the concentrations of HRP, H_2_O_2,_ and PHEA-HP-2. The results of in vitro cytotoxicity against L929 cells and hemolysis experiment indicated that PHEA-HP-2 was safe for the human body. Furthermore, MB-loaded hydrogel beads based on PHEA-HP-2 were successfully prepared using a coaxial needle. Results showed that the viscosity of dimethicone had a significant effect on whether regular spherical hydrogel beads could be formed, and the diameters of the hydrogel beads decreased with increasing the flow rate of the outer phase. Importantly, the diameters of hydrogel beads had a high correlation with the flow rate of outer phase in the range of 640–1440 μL/min. This work not only provides a poly(amino acid) derivative for the preparation of in situ hydrogel, but also provides a new preparation strategy, which has important guiding significance for the design and development of hydrogel beads for applications closely related to the human body, such as wound repair or topical drug delivery for skin.

## 4. Materials and Methods

### 4.1. Materials

3-(4-Hydroxyphenyl) propionic acid (HP, 98%), ethanolamine (99%), 1-Ethyl-3-(3-dimethyllaminopropyl) carbodiimide hydrochloride (EDC·HCl), 4-dimethylaminopyridine (DMAP), and *L*-aspartic acid (98%) were purchased from Shanghai Adamas Reagent Co., Ltd. (Shanghai, China) and used as received. Dimethicones of various viscosities were purchased from Shanghai Aladdin Biochemical Technology Co., Ltd. (Shanghai, China). Hydrogen peroxide (H_2_O_2_, 30 wt.% in H_2_O) was purchased from Sinopharm Group (Shanghai, China). Horseradish peroxidase (HRP, RZ > 2.0, 150 units/mg) was obtained from Shanghai Macklin Biochemical Technology Co., Ltd. (Shanghai, China). Other reagents and solvents were of analytical grade and used without further purification.

### 4.2. Synthesis of PSI and PHEA

Poly(succinimide) (PSI) was prepared by acid-catalyzed polycondensation of *L*-aspartic acid under reduced pressure at 180 °C according to previous work with slightly modification [46]. Briefly, 30 g *L*-aspartic acid and 15 g o-phosphoric acid were added to a 1 L flask and reacted at 180 °C for 4 h using a rotary evaporator to remove by-product water. Then, the resulting product was washed directly with deionized water until neutrality to remove o-phosphoric acid and unreacted *L*-aspartic acid, and the collected solid was air-dried at 95 °C for 24 h for pre-drying. Finally, after vacuum drying and pulverization, the white solid product PSI (20.5 g) was obtained with the yield of 93.7%.

Poly(α,β-[*N*-(2-hydroxyethyl)-D,L-aspartamide]) (PHEA) was synthesized by the ring-opening reaction of PSI with excess ethanolamine at room temperature for 4 days in DMF [47], and purified by dialysis (MWCO 3.5 kDa) against deionized water for 5 days to remove DMF and unreacted ethanolamine. After lyophilization, a white cotton-like PHEA was collected.

### 4.3. Synthesis of PHEA-HP

A total of 1.26 g PHEA (8.0 mmol polymer units) and varying amounts of 3-(4-Hydroxyphenyl) propionic acid (1.0 equiv.) were dissolved in 20.0 mL of DMF. After dissolution, EDC·HCl (1.2 equiv.) and DMAP (1.0 mmol) were added as the coupling agent and catalyst, respectively. After the reaction was carried out at room temperature for 24 h, the obtained solution was added to a dialysis tube (MWCO 3.5 kDa) and dialyzed against deionized water for one week to remove DMF. Then, the dialysate was centrifuged at 8000 rpm (3.0 min) and filtered (0.22 um syringe filter) to remove water-insoluble impurities to obtain a clear and transparent solution. Finally, the white flocculent product PHEA-HP (about 1.1 g) was collected after lyophilization.

### 4.4. Characterizations

Fourier transformed infrared (FT-IR) spectra of PSI, PHEA, and PHEA-HP were recorded with Nicolet 6700 spectrometer (Thermo Fisher Scientific, Hampton, NH, USA). UV absorption spectra of 3-(4-Hydroxyphenyl) propionic acid, PHEA, and PHEA-HP were measured by a UV-2550 UV/Vis spectrometer (Shimadzu, Kyoto, Japan). ^1^H nuclear magnetic resonance (^1^H NMR) spectra of PHEA and PHEA-HP were determined by AVANCE III HD spectrometer (400 MHz) (Bruker, Billerica, MA, USA) in D_2_O or DMSO-*d*_6_.

The molecular weights and molecular-weight distribution of PHEA were evaluated by a GPC system consisting of a Shimadzu GPC-20A (Kyoto, Japan), an Shodex RI-201H refractive index detector (Showa Denko K.K., Tokyo, Japan), an Agilent PLgel 5 µm MiniMIX-C guard column (50 × 4.6 mm, Santa Clara, CA, USA), and an Agilent PLgel 5 µm MIXED-C column (300 × 7.5 mm, Santa Clara, CA, USA). Calibration was achieved using a polystyrene EasiVial Pre-weighed Calibration Kit (PS-M, PL2010-0301, range of nominal M_p_: 162-400,000) (Agilent, Santa Clara, CA, USA). DMF containing 10 mM LiBr was used as the eluent at a flow rate of 0.5 mL/min at 35 °C.

### 4.5. Formation of PHEA-HP-Based Hydrogels

PHEA-HP was dissolved in 100 μL deionized water, and divided into two equal parts (50 μL each). A total of 50 μL HRP solution was added to one part, which was recorded as solution A. A total of 50 μL H_2_O_2_ solution was added to the other part, which was recorded as solution B. Then, two solutions A and B were, respectively, drawn with two syringes and injected into a 1-mL glass bottle through a mixing tube. The polymer concentration was finally controlled at 3.0 wt.% or 6.0 wt.%.

The mixing solutions were considered to be in a gel state if no flow was visually observed within 20 s after inverting the vial. The gelation time of PHEA-HP was determined using the vial-tilting method [37].

For rheological measurements, 500 μL of PHEA-HP/HRP solution (solution A) and 500 μL of PHEA-HP/H_2_O_2_ solution (solution B) were quickly transferred to the parallel plate (plate diameter = 30 mm, fixed gap = 1000 μm) of the rheometer (DHR-2, TA Instruments, New Castle, DE, USA) through the mixing tube with a syringe. The test was performed with a frequency of 1 Hz and 1% strain at 25 °C. The storage modulus (G′) and loss modulus (G″) of the sample were recorded as a function time (10 min). A frequency sweep test was also performed with a frequency range of 0.1 to 5.0 Hz.

### 4.6. Cytotoxicity Assay

The cytotoxicity of PHEA-HP was evaluated against mouse fibroblast L929 cell lines (Shanghai Institute of Biochemistry and Cell Biology, Shanghai, China) by CCK-8 assay. The cells were seeded into a 96-well plate at a density of 10,000 cells/well and allowed to grow at 37 °C under a humidified atmosphere of 95% air and 5% CO_2_ for 24 h. Thereafter, the medium was replaced with 100 μL of fresh medium, and 100 μL solution of PHEA-HP was added (final polymer concentrations ranging from 0.05 to 500 μg/mL). The cells were incubated for 48 h at 37 °C. Next, 100 μL culture medium containing 10 μL of CCK-8 reagent was added into each well for a further 4 h incubation at 37 °C. The absorbance was detected at 450 nm with a Multiskan GO microplate spectrophotometer (Thermo Fisher Scientific, Hampton, NH, USA). Each experiment was carried out in quadruplicate. Cell viability was calculated with the Equation (3):

(3) $$Cell\;viability(\%)=\frac{{OD}_{sample}-{OD}_{blank}}{{OD}_{control}-{OD}_{blank}}\times 100\%$$

where OD_sample_ is the absorbance of solution with cells treated by PHEA-HP, OD_control_ is the absorbance for untreated cells (without PHEA-HP), and OD_blank_ is the absorbance without cells.

### 4.7. Hemolysis Experiment

The hemolysis property of PHEA-HP was evaluated with red blood cells (RBC) dispersion in PBS (5%) collected from New Zealand white rabbits. PHEA-HP solution with various concentrations (400 μL) was mixed with RBC dispersion (600 μL) in the centrifuge tubes and incubated at 37 °C with shaking for 1 h. The PBS buffer and 1% Triton X-100 were used as a negative control (NC) and positive control (PC), respectively. Then, the tubes were centrifuged at 1000 rpm for 10 min and the absorbance of the supernatants was determined by a microplate reader at 540 nm to calculate the hemolysis ratio by the following Equation (4):

(4) $$Hemolysis\;ratio(\%)=\frac{AS-AN}{AP-AN}\times 100\%$$

where AS was the absorbance of the PHEA-HP solutions, AP was the absorbance of PC, and AN was the absorbance of NC.

### 4.8. Preparation of Hydrogel Beads

Two syringes filled with 1.0 mL of solution A (PHEA-HP/HRP aqueous solution) containing 0.5 mg/mL MB and solution B (PHEA-HP/H_2_O_2_ aqueous solution), respectively, were installed in pump A (TYD02-04 four-channel syringe pump, Baoding Lead Fluid Technology Co., Ltd., Baoding, China). Solutions A and B flowed into the mixer through the pipeline and were mixed to serve as the inner dispersed phase. The syringe containing dimethicone as the outer continuous phase was installed in pump B (TYD01-01 signal-channel syringe pump, Baoding Lead Fluid Technology Co., Ltd., China). Driven by two syringe pumps, the inner phase and the outer phase flowed into a coaxial needle (outer continuous phase tube: outer diameter = 1500 μm, inner diameter = 1100 μm; inner dispersed phase tube: outer diameter = 700 μm, inner diameter = 400 μm) through two different interfaces, and then flowed into the dimethicone of the collection container and completed gelation during the sedimentation process to form PHEA-HP-based hydrogel beads. Methylene blue was added to solution A for easy observation. The flow rate of pump A was fixed at 320 μL/min, and the flow rate of pump B varied within a range of 320–1440 μL/min. The resulting hydrogel beads were photographed using a Murzider MSD-2000 digital camera (Murzider Technology Co., Ltd., Dongguan, Guangdong, China) attached to a Soptop BH200M microscope (Sunny Optical Technology (Group) Co., Ltd., Yuyao, Zhejiang, China), and the diameters of the hydrogel beads were analyzed by the software Murzider 4.11 (Murzider Technology Co., Ltd., Dongguan, Guangdong, China).

## Figures and Tables

**Table 1:** Synthesis and characterization of PHEA-HP.

Sample	Feed Ratio of HP to the Polymer Units of PHEA (mol.%)	Percentage of Introduced HP to the Polymer Units of PHEA (mol.%) ^1^	Efficiency (%)	Yield (%)
PHEA-HP-1	30	4.8	16.0	69.3
PHEA-HP-2	50	7.9	15.8	56.7
PHEA-HP-3	80	8.7	10.9	48.9
PHEA-HP-4	100	9.2	9.2	45.3

^1^ Calculated from the ^1^H NMR spectra of PHEA-HP in DMSO-*d*_6_.

## Data Availability

The original contributions presented in this study are included in the article/Supplementary Materials. Further inquiries can be directed to the corresponding author.

## Supplementary Materials

The following supporting information can be downloaded at: https://www.mdpi.com/article/10.3390/gels11020093/s1, Figure S1: ^1^H NMR spectrum of PHEA in D_2_O. Figure S2: FT-IR spectra of PHEA-HP-1, PHEA-HP-2, and PHEA-HP-3 in KBr. Figure S3: The storage modulus (G′) and loss modulus (G″) of PHEA-HP-1/HRP/H_2_O_2_ solutions as a function of time at 25 °C (PHEA-HP-1: 6.0 wt.%, H_2_O_2_: 5.0 mM).
