# Supplementary material for: Enzymatically Cross-Linked Hydrogel Beads Based on a Novel Poly(aspartamide) Derivative
Source: Gels. 2025 Jan 26;11(2):93. doi: 10.3390/gels11020093 (PMC11854777; doi:10.3390/gels11020093)
Supplement: Supplementary file 1 [file gels-11-00093-s001.zip › gels-3428878-supplementary.pdf]

Supplementary Materials

Enzymatically Cross-linked Hydrogel Beads based on a Novel Poly(aspartamide) Derivative

Wenzhuo Hou [1], Hui Yi [1] and Guangyan Zhang [1,2,*]

[1] School of Materials and Chemical Engineering, Hubei University of Technology, Wuhan 430068, China; houwenzhuohbut@163.com (W.H.); yihuihbut@163.com (H.Y.)
[2] Hubei Provincial Key Laboratory of Green Materials for Light Industry, Hubei University of Technology, Wuhan 430068, China
* Correspondence: gyzhang@hbut.edu.cn (G.Z.)

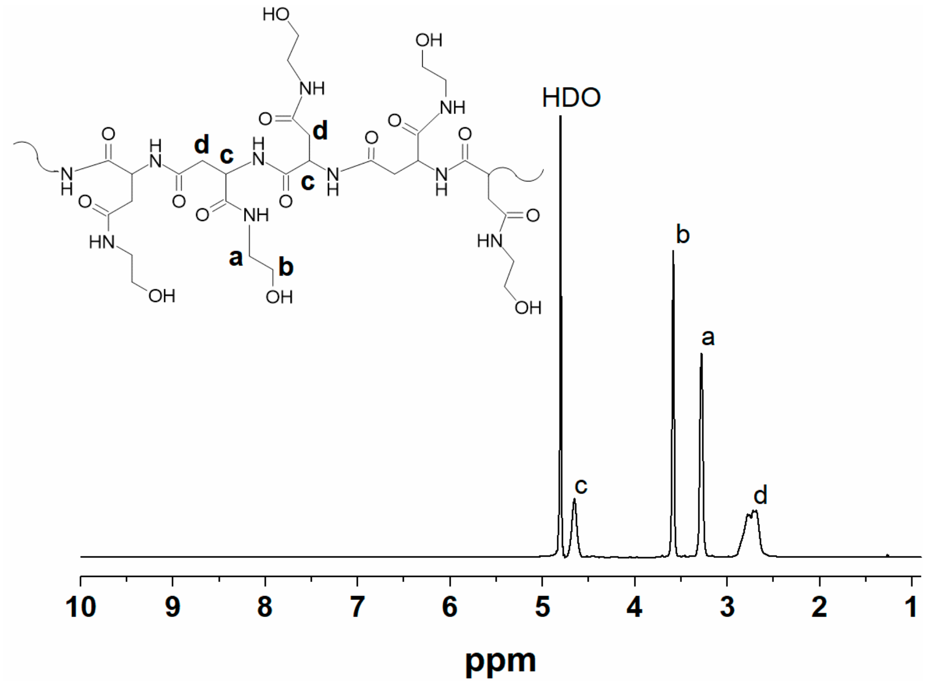

Figure S1. ^1H NMR spectrum of PHEA in D_2O.

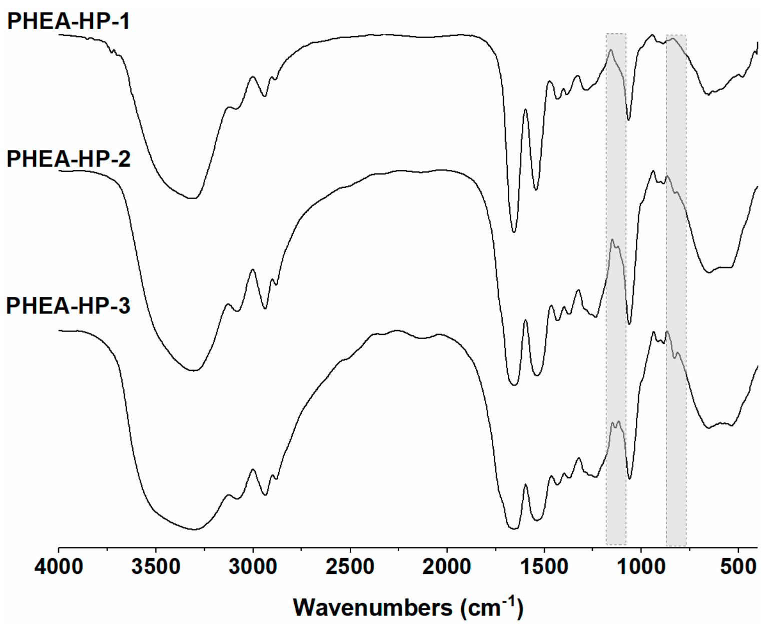

Figure S2. FT-IR spectra of PHEA-HP-1, PHEA-HP-2 and PHEA-HP-3 in KBr.

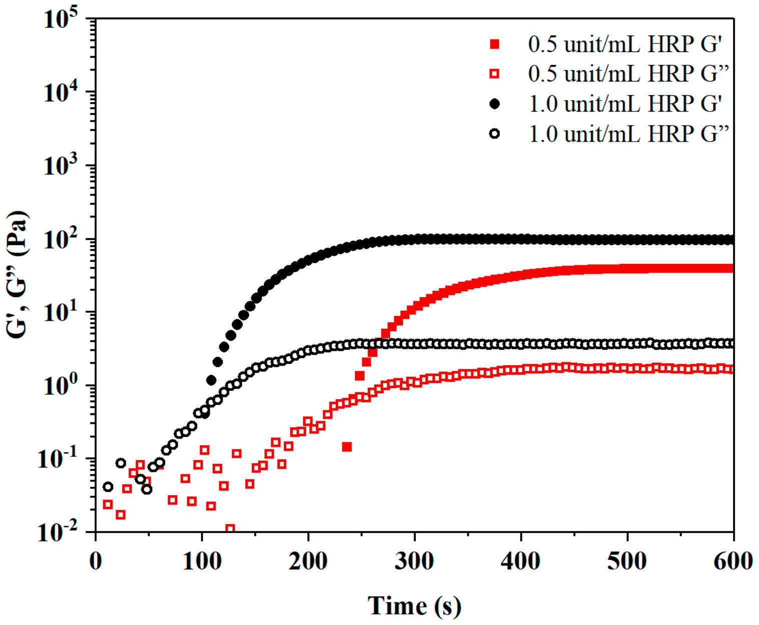

Figure S3. The storage modulus (G') and loss modulus (G") of PHEA-HP-1/HRP/H_2O_2 solutions as a function of time at 25°C (PHEA-HP-1: 6.0 wt.%, H_2O_2: 5.0 mM).
